# The impact of breastfeeding on the preterm infant’s microbiome and metabolome: a pilot study

**DOI:** 10.1038/s41390-024-03440-9

**Published:** 2024-08-13

**Authors:** Keriann Schulkers Escalante, Shiyu S. Bai-Tong, Sarah M. Allard, Gertrude Ecklu-Mensah, Concepcion Sanchez, Se Jin Song, Jack Gilbert, Lars Bode, Pieter Dorrestein, Rob Knight, David J. Gonzalez, Sydney A. Leibel, Sandra L. Leibel

**Affiliations:** 1 Department of Pediatrics, University of California San Diego, Rady Children’s Hospital, La Jolla, CA USA; 2 Department of Pediatrics, University of California San Diego, La Jolla, CA USA; 3 Department of Pharmacology and the Skaggs School of Pharmacy and Pharmaceutical Sciences, University of California San Diego, La Jolla, CA USA; 4 Center for Microbiome Innovation, University of California San Diego, La Jolla, CA USA; 5 Department of Pediatrics and Scripps Institution of Oceanography, University of California San Diego, La Jolla, CA USA; 6 Department of Pediatrics, Larsson-Rosenquist Foundation Mother-Milk-Infant Center of Research Excellence (MOMI CORE), and the Human Milk Institute (HMI), University of California San Diego, La Jolla, CA USA; 7 Collaborative Mass Spectrometry Innovation Center, University of California San Diego, La Jolla, CA USA

## Abstract

**Background:**

Human milk is unquestionably beneficial for preterm infants. We investigated how the transition from tube to oral/breastfeeding impacts the preterm infants’ oral and gut microbiome and metabolome.

**Methods:**

We analyzed stool, saliva, and milk samples collected from a cohort of preterm infants enrolled in the MAP Study, a prospective observational trial. The microbiome and metabolome of the samples were analyzed from 4 longitudinal sample time points, 2 during tube feeds only and 2 after the initiation of oral/breastfeeding.

**Results:**

We enrolled 11 mother-infant dyads (gestational age = 27.9 (23.4–32.2)) and analyzed a total of 39 stool, 44 saliva, and 43 milk samples over 4 timepoints. In saliva samples, there was a shift towards increased *Streptococcus* and decreased *Staphylococcus* after oral feeding/breastfeeding initiation (*p* < 0.05). Milk sample metabolites were strongly influenced by the route of feeding and milk type (*p* < 0.05) and represented the pathways of Vitamin E metabolism, Vitamin B12 metabolism, and Tryptophan metabolism.

**Conclusion:**

Our analysis demonstrated that the milk and preterm infant’s saliva microbiome and metabolome changed over the course of the first four to 5 months of life, coinciding with the initiation of oral/breastfeeds.

**Impact:**

- The microbiome and metabolome is altered in the infant’s saliva but not their stool, and in mother’s milk when feeds are transitioned from tube to oral/breastfeeding.
- We assessed the relationship between the gut and oral microbiome/metabolome with the milk microbiome/metabolome over a longitudinal period of time in preterm babies.
- Metabolites that changed in the infants saliva after the initiation of oral feeds have the potential to be used as biomarkers for disease risk.

## Introduction

Infants born preterm are at higher risk of morbidity and mortality than those born at term.^1^ It is well established that early colonization of the infant’s digestive tract has a significant impact on long term health outcomes.^2–6^ Preterm infants have reduced bacterial microbiota diversity and an abnormal intestinal colonization pattern compared to their term counterparts, which increases their susceptibility to disease.^2^

Human milk (HM) provides numerous benefits to newborns and infants, including helping to establish a normal gut microbiome, decreasing the risk of atopy,^7,8^ and improving neurodevelopmental outcomes.^9^ HM is particularly protective for the preterm infant as it can decrease the risk of necrotizing enterocolitis and sepsis.^10–14^ and is impacted by the route of oral feeds. A recent study showed that in moderately preterm infants (32–34 weeks gestation), the HM microbiome composition changed following the start of breastfeeding, altering the infants’ saliva and gut microbiome with a higher proportion of *Bifidobacterium* and a lower proportion of *Pseudomonas* in both fecal and saliva samples.^15^

The byproduct of microbes are metabolites and breastfeeding produces a potent combination of stimulatory and inhibitory metabolites such as reactive oxygen species in the infant’s saliva that regulate early oral–and gut–microbiota. In turn, the detection of infant saliva by the mammary cells has been shown to change the composition of the HM.^16^

While direct breastfeeding is ideal for establishing a healthy microbiota, most preterm infants receive milk feeds through an orogastric/nasogastric (OG/NG) tube due to the lack of oral feeding developmental skills. A recent study showed that the gastric contents of preterm infants who were fed HM via an OG/NG tube were colonized with Proteobacteria and Firmicutes and the high-risk sepsis-associated bacteria *Enterobacter faecalis* ST64.^2^ This suggests that feeding via enteral feeding tubes alone can have a negative impact on establishing a healthy gut microbiota.

We hypothesized that exposure to milk orally, including breastfeeding, would increase the diversity of the preterm infants oral and stool microbiome, which would subsequently alter the microbiome and metabolome of the pumped HM. The present study aims to utilize a cohort of preterm infants born at or before 34 weeks GA, enrolled in the Microbiome, Atopic Disease, and Prematurity (MAP) Study.^17^ that were initially fed with tube feeds then transitioned to oral/breastfeeding. Samples of the stool, saliva, and milk from 4 longitudinal time points during their Neonatal Intensive Care Unit (NICU) stay (2 prior to and 2 after the initiation of oral feeds) were analyzed and compared with the clinical data of the infant and mother.

## Materials and methods

### Ethics statement

The University of California San Diego Institutional Review Board approved all study protocols (IRB approval #181711). All research, experiments and data analysis were performed in accordance with the University of California guidelines and regulations. Participant parents/guardians provided written, informed consent prior to enrollment in this research project. Furthermore, the “MAP (Microbiome, Atopic Disease, Prematurity) Study” was registered in ClinicalTrials.gov database (NCT04835935).

### Study population and design

#### MAP Study

This study used samples collected from a cohort of preterm infants from the MAP study, a prospective observational study conducted from June 2019 to September 2020 at two level III NICUs at University of California San Diego (UCSD) and Rady Children’s Hospital in San Diego, California. Infants were enrolled if they were born at or before 34 weeks gestation without any prenatally diagnosed congenital anomalies. A total of 46 infants (including 11 pairs of twins) were enrolled.^17^ Stool, saliva, and milk feed samples were collected weekly during their NICU stay. Milk samples were collected from the feeding syringe or bottle before or after feeding events. These included unfortified HM, fortified HM, and formula. Maternal demographic information was collected, including maternal dietary history during pregnancy, family history of atopic disease, and antenatal courses. Infant demographics collected included birth history and clinical course data.

#### Tube vs. oral/breastfeeding cohort

From the cohort of 46 preterm infants enrolled in the MAP study, we selected 11 infants who received HM throughout their stay in the NICU including breastfeeding. For each mother-infant pair, we identified four sample time points: two before the initiation of oral feeds and two after. At each time point, stool, saliva, and milk samples were collected for paired metagenomic and metabolomic analysis.

### Measurements

#### Metagenomics

The University of California Microbiome Core performed nucleic acid extractions using previously published protocols.^18^ Briefly, extractions and purifications were performed using the MagMAX Microbiome Ultra Nucleic Acid Isolation Kit (Thermo Fisher Scientific) and automated on KingFisher Flex robots (Thermo Fisher Scientific). Blank controls and mock communities (Zymo Research Corporation) were included per extraction plate, which were carried through all downstream processing steps. Input DNA was quantified using a PicoGreen fluorescence assay (Thermo Fisher Scientific) and metagenomic libraries were prepared with KAPA HyperPlus kits (Roche Diagnostics) following manufacturer’s instructions and automated onEpMotion automated liquid handlers (Eppendorf).

Sequencing was performed on the Illumina NovaSeq 6000 sequencing platform with paired-end 150 bp cycles at the Institute for Genomic Medicine (IGM), UC San Diego. 10 milk samples were sequenced before the full sample set to ensure adequate quality due to low biomass. All milk samples were then extracted and sequenced as described above. An initial analysis was performed to confirm that the samples and mock communities from each run were comparable, and then reads corresponding to the same samples were merged in the final analysis.

Raw metagenomic sequences were quality filtered and trimmed using fastp v 0.22,^19^ and human and PhiX reads were filtered using minimap2 version 2.22.^20,21^ and converted to FASTQ format using SAMtools version 1.15.1.^22^ Reads were aligned against the Web of Life database.^23^ (release 2, human genome included.^24^) using Bowtie2.^25^ and classified with the Woltka pipeline.^26^ (v. 0.1.5), resulting in a table of Operational Genomic Units (OGUs) and per-sample genome coverage profiles. Sequences matching the human reference genome and sequences matching genomes with less than 5% coverage per sample were removed from downstream analysis.

For across-sample type analysis, controls were removed, and all samples were rarefied to 121,270 reads. Specific rarefaction levels were chosen based on rarefaction curves and sequencing depth for within-sample type analysis (stool: 1,288,710; milk: 2990; and saliva: 47,195).

Faith’s Phylogenetic Diversity.^27^ over time was assessed with generalized linear mixed effects models for each sample type using the lme4 package.^28^ in R version 4.0.2.^29^ Using lme4, we fit linear mixed-effects models to the Faith’s phylogenetic diversity data within each sample type, accounting for the subject as the random effect in the null model. We compared the fit between models using ANOVA through lmerTest, with several other metadata categories used as fixed effects individually and in conjunction, to identify which were required for model performance. Differences in bacterial community composition were calculated between sample groups using Unweighted and Weighted UniFrac distance matrices,^30^ and statistical differences were assessed with permutational multivariate analysis of variance (PERMANOVA). Figures were generated in QIIME2.^31^ and R version 4.0.2.^29^ with tidyverse.^32^ and ggplot2.^33^ packages.

Differential abundance analysis to identify log fold change of feature ranks across the different sampling time points was performed with songbird multinomial regression analysis.^34^ A pseudocount of 1 was added to the OGUs table. Parameters optimized for the model (parameters: –formular = time point, –epochs = 100,000, differential-prior = 0.5, –summary interval = 1, –min-sample-count = 1000, –min-feature-count = 10) was determined by cross-validation (CV) minimization. The model fit was compared by a pseudo Q-squared value, defined as 1 − model CV/baseline CV) value, where a Q-squared value of >0 represents a good model fit.

Log ratios of the resulting differentials were explored and visualized in Qurro.^35^ The 20 highest and 20 lowest ranked microbes differentially associated with time points 1 and 4, as well as the most relatively abundant bacterial genera informed the taxonomic groups to compute the log ratios. The smallest sets of nonoverlapping features that provided meaningful differences between the first and last time points for the different sample types were used and compared across all time points. Statistical significance between log ratios over time was assessed with linear mixed effect models (lme4 v 1.1–33) with Tukey method for adjusting multiple comparisons.

#### Metabolomics

Saliva and stool swabs were transferred to sterile microfuge tubes and 400 μl of LCMS grade water was added to each sample. Samples were then shaken on a Qiagen TissueLyser II for 5 min at 25 Hz, then subsequently spun down for 5 min at max speed. Swabs were removed from each tube, then 400 μl of 100% methanol was added to each tube. Samples were spun down again for 5 min at max speed, then samples were incubated at −20C for 30 min. 200 μl of each supernatant was then transferred to a 96w plate for centrifugal lyophilization. 50 μl of each milk sample was transferred to a 96-well deep well plate, then 400 μl of a 3:1 MeOH:MTBE solution was added to each sample. Samples were sonicated for 5 min in a water bath sonicator, then centrifuged for 15 min at 1800 rpm. 300 μl of each supernatant was then transferred to a 96w plate for centrifugal lyophilization.

Untargeted LC-MS/MS analysis was performed on a Thermo Q-Exactive Mass Spectrometer coupled to a Thermo Vanquish HPLC system (Thermo Fisher Scientific). The chromatographic analysis was carried out on a Polar C18 100 A LC Column 100 × 2.1 Mmm (Catalog no. 00D-4759-AN). The mobile phase comprised Optima LC-MS grade water with 0.1% formic acid (phase A) and Optima LC-MS grade acetonitrile with 0.1% formic acid (phase B) with flow rate set to 0.5 mL/min. The data were collected in positive ion mode. Raw data were converted to.mzXML format using Thermo Fisher data analysis software and uploaded to the UCSD MassIVE data repository.

Thermo.raw files were first converted to mzML using MSConvert (3.0.22278-1d7bb32). mzML files were processed by sample type (stool, saliva, milk) using MzMine (3.5.0).^36^ to identify metabolite features. Stool samples were processed in combination with their respective QC Files. Parameters for individual sample metabolite feature identification were as follows: Mass Detection (MS1 Noise Level:1.0E3, MS2 Noise Level: 5.0E2), Feature Detection through ADAP Chromatogram Builder (min group size in # of scans = 4, group intensity threshold=3000, min highest intensity = 1000, m/z tolerance = 0.005 Da or 10 ppm), Feature Detection Chromatogram Resolving (MS/MS scan pairing with RT Tolerance = 0.10 min and MS1-MS2 precursor tolerance = 0.0100 m/z; Local min search used with chromatographic threshold = 90%, min RT range 0.50 min, min relative height 0.01%, min absolute height 1000, min ratio of peak top/edge = 1.7, peak duration 0.05–1 min, and min # data points = 4), 13C Isotope filter (m/z tolerance = 0.01 m/z, RT tolerance = 0.30 min, and maximum charge = 5). Parameters for metabolome feature bucket table were as follows: Join Aligner (m/z tolerance = 0.01 m/z, m/z weight = 80, RT tolerance = 0.30 min, RT weight = 20), Feature list filtering (at least 2 peaks per row), and Gap Filling (intensity tolerance = 10%, m/z tolerance = 0 m/z, RT tolerance = 0.4). Metabolite feature tables were then exported using the Global Natural Products Social molecular networking (GNPS) FBMN option to generate required files for online GNPS FBMN analysis.

Feature annotations were done through the GNPS Feature-Based Molecular Networking workflow (Version 28.2).^37^ MS2 MGF files were exported from MzMine along with a feature quantification table and imported to the FBMN workflow. Spectral library files were set to search all GNPS Spectral Libraries (speclibs). Features with annotation were then appended to the feature quantitation table exported from MzMine.

Metabolite features were first normalized to the intensity of value of the internal standard, sulfamethazine, in each sample and then multiplied by 1E6. Missing values (with peak intensities of 0) in metabolite features were set to NA. Then, features with more than 20% missing values per group (time point) were removed from analysis. Missing values in remaining features were imputed using K-Nearest Neighbor (KNN) imputation using the “impute” R package (1.68.0). Intensity values were then log2 transformed.

Principal coordinate analysis (PCoA) was conducted with metabolite features for each sample type (stool, saliva, milk) using Bray-Curtis distance calculation in the “stats” R package. Here, batch effects were discovered for stool metabolomics samples based on MS acquisition methods. Stool samples were thus excluded from further analysis. PERMANOVA analysis was conducted using categorical metadata and metabolite features using Bray-Curtis distance calculation in the “vegan” R package. Binary comparisons between time points were done through the R ‘stats‘ package using Student’s *T*-test with adjusted *p* values. Volcano plots were created in GraphPad Prism (9.5.0). All other plots were made using the “ggplot” package in R. MetaboAnalyst (5.0).^38,39^ was used for metabolite functional enrichment analysis using MS peaks ranked by Student’s *t* test *p* values. A p-value cutoff of 0.05 was used for the mummichog algorithm.

## Results

### Characteristics of infants and mothers in the cohort

This study analyzed samples collected from a cohort of preterm infants from the MAP study.^17^ The clinical data from the cohort of 11 infant-maternal dyads showed an average maternal age of 29 years; 100% of mothers received prenatal betamethasone, 73% of mothers underwent a cesarean section, and 45% of mothers experienced premature prolonged rupture of membranes and received latency antibiotics (Table 1). The average GA at birth was 27.9 weeks; 64% of infants were intubated and received surfactant, 73% of infants received antibiotics for ≥2 days, and 18% received an antifungal (Table 2). Further characteristics including preterm morbidities are found in Supplementary Table 1.

**Table 1:** Maternal demographics, history, and antenatal course.

Characteristics		Maternal-Infant dyads *n* = 11	
Maternal Age, average year (range)		29 (22–41)	
Mothers with a twin gestation pregnancy, *n* (%)		5 (45%)	
Betamethasone use, *n* (%)		11 (100%)	
C-section, *n* (%)		8 (73%)	
Prenatal Betamethasone, *n* (%)		11 (100%)	
PPROM, *n*		5 (45%)	
Latency Antibiotic Use, *n* (%)		5 (45%)	
Maternal Chorioamnionitis, *n* (%)		3 (27%)	
Antibiotic given for chorioamnionitis, *n* (%)		3 (27%)	
Other antibiotic use during pregnancy, *n* (%)		6 (55%)	
Pregnancy Complications, *n* (%)	Diabetes (Gestational or Type I/II)		2 (18%)
	Hypertension (Chronic or Gestational)		2 (18%)
	Pre-eclampsia or HELLP		3 (27%)
	Abruption		2 (18%)
Ethnicity (maternal), *n* (%)	White		4 (36%)
	Hispanic or Latino		5 (46%)
	White/black mixed		2 (18%)
	Other		0

*PPROM* premature prolonged rupture of membrane, *HELLP* hemolysis, elevated liver enzymes, low platelet count.

**Table 2:** Infant demographics, birth history, and NICU Course.

Characteristics	Maternal-Infant dyads *n* = 11
Gestational Age, average weeks (range)	27.9 (23.4–32.2)
Male infants, *n* (%)	4 (36%)
Number of infants who are a twin, *n*	5
Pair of twins, *n*	2
Average weight at birth, g (range)	1113 (575–1855)
Median APGAR at 1 min, (range)	4 (1–8)
Median APGAR at 5 min, (range)	8 (3–9)
Number of infants who were intubated, *n* (%)	7 (64%)
Average intubation duration, days (range), *n* = 7	8 (1–23)
Surfactant administration, *n* (%)	7 (64%)
Number of infants who received systemic steroids, *n* (%)	2 (18%)
Number of infants exposed to postnatal antibiotics >2 days, *n* (%)	8 (73%)
Number of infants exposed to postnatal antifungal, *n* (%)	2 (18%)
Number of infants required antacid, *n* (%)	1 (9%)

All infants received HM or donor HM, bovine-based HM fortifier, and/or preterm formula (Supplementary Table 2) based on the unit’s feeding policy, infant’s clinical status, and infant’s corrected GA (CGA) at the time of sample collection. All infants followed the NICU’s standard feeding policy which bases enteral feeding progression on the infant’s weight at birth. Infants initially received unfortified HM (with donor HM as supplementation if HM not available or insufficient) and were fortified to 22 kcal/oz at 80 ml/kg/day and 24 kcal/oz at 120 ml/kg/day, with further fortification added per individual need. They were allowed to participate in non-nutritive breastfeeding when >30 weeks CGA as long as they were considered medically stable independent of the type of non-invasive respiratory support they were receiving. Oral feeding (via breastfeeding and/or bottle) was initiated when the infant was on minimal respiratory support (low flow nasal cannula) and showing oral feeding cues, which was typically around 34 weeks CGA. When starting oral/breastfeeding, each infant followed a standardized protocol with a combination of oral and tube feeds, until they reached full oral feeds. For each subject, 4 longitudinal samples were collected, two prior to the start of oral feeds and two after starting oral feeds. On average, timepoint 1 was collected at 2 weeks of life, timepoint 2 at 5 weeks of life, timepoint 3 at 9 weeks of life, and timepoint 4 at 12 weeks of life (Table 3). A total of 39 stool, 44 saliva, and 43 milk samples were analyzed. Additional information on infant’s weight gain on tube versus oral feeds is available in Supplementary Table 3.

**Table 3:** Sample time points collected for stool, saliva and milk.

	Sample time point	Average number of weeks of life at time of collection *(range)*	Average of days on oral feeds (bottle and/or breast) (*range*)
Before oral feeds	1	2 weeks (1–4)	N/A
	2	5 weeks (2–10)	N/A
After oral feeds	3	9 weeks (5–16)	23 days (9–39)
	4	12 weeks (7–19)	39 days (23–56)

### Transition to oral feeds had the greatest impact on the saliva microbiome diversity

**Fig. 1:**
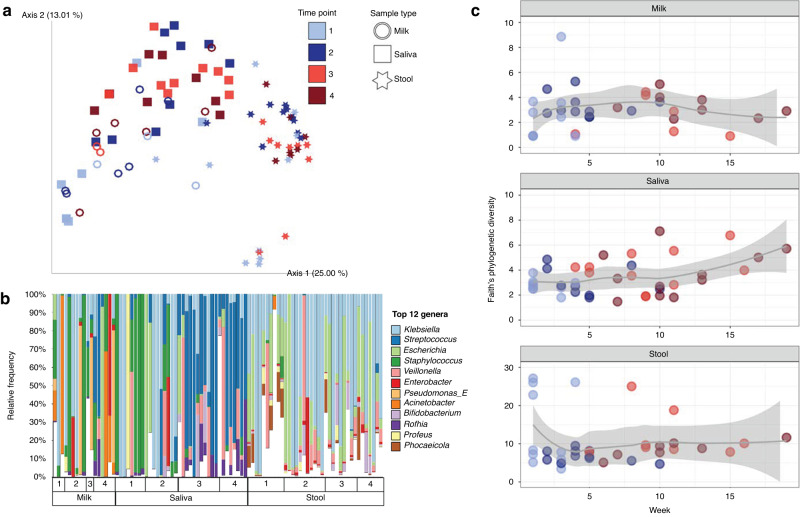
Bacterial communities from milk, saliva, and stool samples over time from mother-infant pairs (*n* = 11) based on shotgun metagenomics data. **a** PCoA plot based on Unweighted UniFrac distance matrix, with rarefaction level of 260,960 sequences per sample (milk, *n* = 17; saliva, *n* = 36; stool, *n* = 37). **b** Top 12 most relatively abundant bacterial genera identified across the dataset are shown for all sample types across time points, rarefied at 121,270 sequences per sample. The remaining other taxa are grouped in white. **c** Alpha diversity by the Faith’s Phylogenetic Diversity metric over time (weeks of life) within each sample type (milk, *n* = 33, rarefied at 2990; saliva, *n* = 39, rarefied at 47,195; stool, *n* = 37, rarefied at 1,288,710).

Microbial communities were significantly influenced by sample type (*R*^2^ = 0.24, *p* = 0.001; all groups distinct, *p* = 0.001), subject (*R*^2^ = 0.14, *p* = 0.001), and time point (*R*^2^ = 0.04, *p* = 0.001), with significant interactions between all these factors (Fig. 1a, PERMANOVA on Unweighted UniFrac distances). Statistical findings were comparable when using weighted UniFrac, which incorporates relative abundance information.

The highest microbial diversity, as measured by Faith’s Phylogenetic Diversity, observed features, and Shannon diversity, was detected in stool (*p* < 0.05 compared to milk and saliva). Milk and saliva were not significantly different. An increase in microbial diversity was observed in saliva samples over time but not the other sample types (Fig. 1c). To examine the relative effects of oral feeding status, week of life, milk type, delivery method (cesarian vs. vaginal), and twin status, we tested mixed effects models within sample types, using null models that included only subject. For saliva samples, there was a significant impact of the transition to oral feeds on saliva alpha diversity (*p* = 0.007), and each subject’s temporal trajectory was personalized (*p* = 0.005, week added in random effect term with subject). For stool, delivery method and week of life had significant effects (*p* = 0.035 and *p* = 0.015, respectively), and milk type, before vs. after oral feeding transition, and the personalized effect of week of life contributed to the best the model (*p* = 0.032). For milk samples (excluding formula), there was no significant trend over time in terms of week of life or oral feeding status, however the model was improved by including milk type (fortified vs. unfortified) as a fixed effect (*p* = 0.035).

### Milk type and transition to oral feeds impacts microbial communities

To tease apart microbial community dynamics, we analyzed the effect of subject and time point on beta diversity within each of the three sample types. In milk samples there were significant effects of both subject (*R*^2^ = 0.32, *p* = 0.006) and time point (*R*^2^ = 0.06, *p* = 0.017, PERMANOVA on Unweighted UniFrac distances), with no significant pairwise differences. Milk types also had significantly different microbial communities, with unfortified and fortified maternal milk hosting unique communities (*R*^2^ = 0.1, *p* = 0.001, PERMANOVA on Unweighted UniFrac distances) (Supplementary Fig. 1). Formula samples had low sequencing depth (1770–2491 sequences per sample, *n* = 4) so were excluded from the overall analysis. The four formula samples contained only 1 species that was confidently assigned after quality filtering, *Streptococcus thermophilus*.

For milk samples (formula excluded), examination of the most differential bacteria between the beginning and ending time points of the study revealed changes in the ratios of certain bacterial groups over time. The most differentially represented top 20 taxa were assigned to *Enterobacter*, *Streptococcus*, *Klebsiella*, *Veillonella*, *Rothia* and *Mesorhizobium* (Fig. 1b); whereas the bottom 20 taxa were within the genera *Stenotrophomonas*, *Serratia*, *Haemophilus*, *Enterococcus*, *Cutibacterium*, *Pseudomonas*, *Staphylococcus* and *Lactobacillus*. The ratio of these two groups increased over time, with significant differences between timepoints 1 and 3 and 1 and 4 (*p* < 0.05; Supplementary Fig. 2) reflecting the transition from tube to oral feeds.

### Stool microbiome did not significantly change after the transition to oral feeds

Stool samples were not significantly influenced by subject or time point for the Weighted or Unweighted UniFrac metrics (*p* > 0.05, PERMANOVA). The type of milk consumed by the participant at the time of sampling did have a significant effect on the microbial communities when relatedness was included but relative abundance was not accounted for (*R*^2^ = 0.12, *p* = 0.036, PERMANOVA on Unweighted UniFrac distances). At the genus level, *Klebsiella* and *Escherichia* were overall the most relatively abundant and prevalent Enterobacteria found in the stool (Fig. 1b). Differential rankings showed that changes in ratio of the relative abundances of *Escherichia* and *Klebsiella* to *Bacteroides* were different only between timepoints 1 and 2 (*p* < 0.05). We further observed significant differences in the ratio of *Bifidobacterium* to *Bacteroides* in stool microbiota between time points 1 and 2 and time points 1 and 4 (*p* < 0.05; Supplementary Fig. 3). No specific taxa were found to vary in prevalence or relative abundance in stool following the initiation of oral feeds (after time point 2).

### *Streptococcus* and *Klebsiella* increase in the salivary microbiome after the transition to oral feeds

**Fig. 2:**
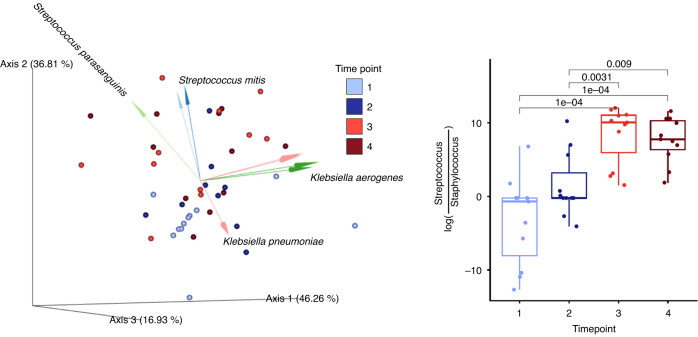
Salivary microbiota is significantly influenced by time point. (Left). Principal component analysis of robust Aitchison distance (RPCA) plot with only saliva samples. The dots are color coded by time points and the biplot arrows are colored by groups of bacterial operational genomic units (OGUs) influencing saliva microbiota community dissimilarity. (Right). Box plots of log ratio of all of the OGUs identified within the genera *Streptococcus* and *Staphylococcus*. Log ratios were calculated and processed using Songbird multinomial regression and visualized in Qurro. Statistical significance was determined by linear mixed effect models, LME, *p* < 0.05.

Microbial communities in saliva samples were influenced most strongly by subject (*R*^2^ = 0.42, *p* = 0.001, PERMANOVA on Unweighted UniFrac distances), time point (*R*^2^ = 0.11, *p* = 0.001, PERMANOVA on Unweighted UniFrac distances), and milk type (*R*^2^ = 0.05, *p* = 0.006, PERMANOVA on Unweighted UniFrac distances), with significant interactions between these three factors (Fig. 2). For time point, there were significant differences when comparing time points 1 and 2 to 3 and 4 (pairwise PERMANOVA, *p* < 0.02). Similar results were observed with Weighted UniFrac distances. Due to a significant interaction between subject and time point in saliva microbiota, we subset the analysis to participants with the complete time series. For this subset, despite the much-reduced sample size (6/11 participants), significant effects remained for both time point (*R*^2^ = 0.144, *p* = 0.022, PERMANOVA on Unweighted UniFrac distances) and subject (*R*^2^ = 0.377, *p* = 0.003, PERMANOVA on Unweighted UniFrac distances). Across all participants, the difference between saliva microbiota collected during tube feeding or during oral feeding time points was in part driven by changes in ratio of the relative abundances of *Staphylococcus* to *Streptococcus*, as well as *Klebsiella* and *Veillonella sp. F0422* (Fig. 2).

### Delivery method in preterm infants does not significantly influence the microbiome

In contrast to studies of full-term infant microbiomes, delivery method (vaginal versus cesarean section) did not exert a significant influence on the stool microbiome (*p* = 0.283, PERMANOVA on Unweighted UniFrac distances), saliva microbiome (*p* = 0.08, PERMANOVA on Unweighted UniFrac distances), or milk microbiome (*p* = 0.335, PERMANOVA).^3,4,6^ Delivery mode did not have a significant effect using Weighted UniFrac for the entire dataset or any individual sample type. A potential effect of delivery mode was identified in the alpha diversity of stool; however sample size was limited. Clinical factors such as age at time of sample, sex, twin status, birth weight, antibiotic use, maternal chorioamnionitis, and number of days intubated were examined, however the low sample size and unique clinical history of each participant did not result in any statistical significance of these factors.

### Transition to oral feeds impacts the milk metabolome

**Fig. 3:**
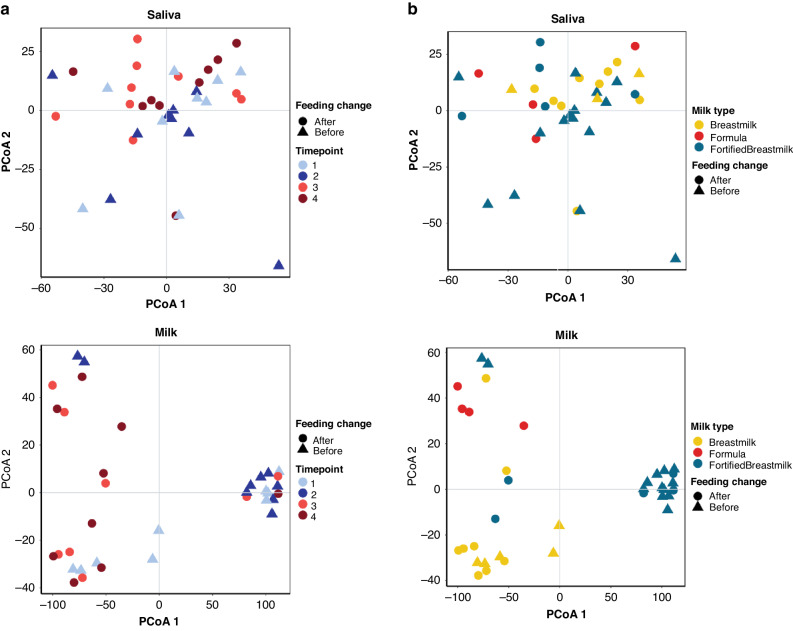
The saliva and milk metabolome are influenced by timepoint, feeding change, and milk type. **a** PcoA using Bray-Curtis Distance of saliva (top) and milk (bottom) samples. Points colored by time point and shape by feeding change group (Before: NG/Tube-feeding; After: Post- NG/Tube-Feeding). **b** PcoA using Bray-Curtis Distance of saliva (top) and milk (bottom) samples. Points colored by milk type and shape by feeding change group (Before: Tube feeding; After: Oral feeding).

To continue our understanding of the impact of tube feeding in the developing infant microbiome we conducted a metabolomics analysis of stool, saliva, and milk samples individually. Milk samples were significantly influenced by time point, feed change, and milk category (PERMANOVA on Bray-Curtis Dissimilarity *p* < 0.05) (Fig. 3). Stool samples were significantly influenced by mass spectrometry acquisition, or batch effect, (PERMANOVA on Bray-Curtis Dissimilarity *p* < 0.001) and were thus excluded from further analysis. Saliva samples displayed no significant influences (PERMANOVA on Bray-Curtis Dissimilarity *p* > 0.05). Time point (Fig. 3a) and milk type (Fig. 3b) influences on saliva and milk samples were visualized using PCoA, where milk samples displayed strong influences based on milk type (HM, fortified milk, or formula).

**Fig. 4:**
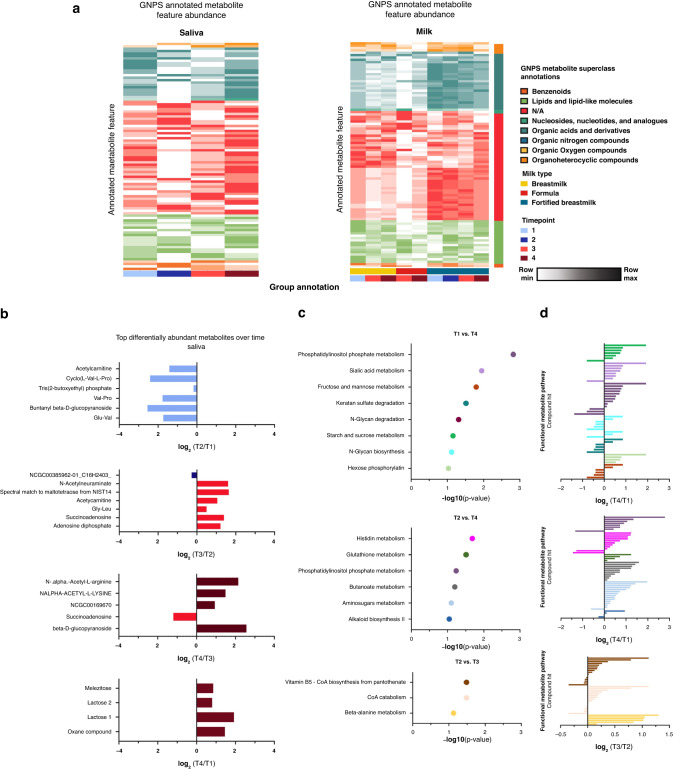
Milk metabolite abundance is altered by milk type, and functional pathway metabolites in saliva are altered across time points. **a** Heatmap of GNPS annotated metabolite features in saliva and milk samples colored by metabolite (row) min to max milk-type across time points. Metabolite average intensity by time point was used for visualization. Column grouping was done by time point and milk type in saliva and milk samples respectively. **b** Bar plots showing differently abundant GNPS annotated metabolites in saliva samples based on timepoint binary comparisons. Significantly altered metabolites are colored by time point in which they are found to be higher in each respective binary comparison. **c** MetaboAnalyst functional pathway analysis across time points for saliva samples. Samples were sorted by *p* value of the respective binary comparisons for pathway analysis. **d** MetaboAnalyst functional pathway compound hits with their respective log2-fold change per comparison. Bars are colored by pathway hit.

As sample time point significantly influenced metagenomic data, we chose to conduct binary comparisons of saliva and milk metabolomics samples by time point, with several significantly altered metabolites found in each binary comparison. We annotated our identified metabolite features through MS/MS annotation using GNPS. Here we found 116 (4.0% annotation rate) annotated saliva and 128 (6.6% annotation rate) annotated milk metabolites. Several GNPS annotated features were significantly altered when comparing by time point (Supplementary Fig. 4). Time point was not independent from milk type, as many participants began on fortified milk and changed to formula or unfortified HM during the course of the study. To further understand changes in GNPS annotated features we plotted average abundance by stratifying milk and saliva samples by milk type and time point, respectively. While saliva samples showed no visible pattern, milk samples showed changes in abundance of metabolites by milk type (Fig. 4a). Specifically, fortified HM had higher abundance of organic acids and derivatives, while unfortified HM had higher abundance of lipids and lipid-like structures. Interestingly, among the altered metabolites in the saliva metabolome were “Lactose products” which were significantly enriched in in timepoint 4 when comparing to timepoint 1 (Fig. 4b).

Because several of the GNPS annotated metabolites lacked functional superclass information, we sought to explore functional metabolic changes using MetaboAnalyst. We conducted a functional pathway analysis using MS1 Peak information for saliva samples for putative metabolite annotation. Functional analysis revealed several significantly altered functional pathways across time points in saliva samples (Fig. 4c). Among the altered pathways were phosphatidylinositol phosphate metabolism, sugar metabolism, amino acid metabolism, and vitamin B5 biosynthesis, where a majority of compound hits were observed to have increased abundance after oral feeding implementation (timepoints 3 and 4) compared to tube feeding time points (timepoints 1 and 2) (Fig. 4d).

## Discussion

The results of this study provide insight into the changes in the diversity and composition of HM and the preterm infants’ saliva microbiome and metabolome as they transition from tube feeds to oral feeds. Significant changes were identified in the microbiome and metabolome of the preterm infant’s saliva and the maternal HM following the initiation of oral feeding. We did not observe significant changes within the stool samples over time.

Previous studies have demonstrated a strong link between HM and the infants’ saliva. The HM microbiome is influenced by numerous factors including the maternal gut and skin, health status, hormonal changes, lactation period, environment, and the infant’s oral cavity.^27,40–42^ Limited evidence exists however on how HM changes after the initiation of oral feeding from tube feeding in preterm infants, a cohort of newborns that are unable to orally feed after birth. Our analysis demonstrated that specific ratios of bacterial genera in HM changed from tube feeds to oral feeds, however significance could not be confirmed due to limited sample size.

In saliva, however, with the initiation of oral feeds, we saw a significant overall increase in the microbial diversity in the preterm infant’s saliva samples. The changes in the genus level community showed a reduction of *Staphylococcus* relative to *Streptococcus* after the initiation of oral feeds. This may reflect the changes seen in the milk samples as well as increased exposure to skin with breastfeeding. This finding is consistent with another recent study by Selway et al., showing a shift from *Staphylococcus* to *Streptococcus* over time in the saliva microbiome of preterm infants.^43^ Al-Shehri et al. previously demonstrated that mixing HM with saliva inhibited in vitro growth of opportunistic pathogens such as *Staphylococcus aureus* and *Salmonella spp*.^16^ Therefore, we hypothesize that exclusive tube feeds may increase the risk of opportunistic pathogens in preterm babies. The implementation of “oral care” or swabbing the cheeks of infants with HM, has reduced the incidence of sepsis in the NICU.^44^

There was an increase in potentially protective bacteria after the initiation of oral feeds in the saliva samples, such as *Veillonella* (Supplementary Fig. 5). *Veillonella* is a common bacterium in the intestines and saliva mucosa and is the most nitrate-reducing bacterium in the oral cavity which acts beneficially as an anti-bacterial.^16^ The abundance of *Veillonella* has been previously found to increase with postnatal age in the gut of preterm infants.^5^

While no significant changes were seen in the diversity of the stool samples between tube and oral feeding, this is likely driven by participant microbiomes being too different from each other to detect common effects without higher replication. In most samples, the stool microbiome was found to be predominantly *Klebsiella* and *Escherichia*. However, we saw a slight relative increase in protective bacteria such as *Bifidobacterium* over time, particularly from timepoint 1 to 2 and from timepoint 1 to 4 (Supplementary Fig. 3). Prior studies have found that *Bifidobacterium* abundance increases over time, correlating to postnatal age.^14,15,45–47^ *Bifidobacterium* is a predominant species in both HM and the infants’ saliva and intestinal mucosa, and is less abundant in the HM of preterm infants compared to their term counterparts.^48^ It plays an important role in reducing the amount of *Streptococcus* bacteria in the mouth and reducing intestinal inflammation.^27,48^ In preterm infants, milk and bovine-based fortifier, was significantly associated with an increased abundance of *Bifidobacterium*.^5^ As such, our results could be reflective of the fact that our infants were more likely to receive unfortified and/or HM fortifiers early in their NICU course (timepoint 1), and more bovine-based fortification later on (timepoints 2–4).

In this study, we also found that the HM and saliva metabolic profiles of preterm infants before oral feeding were significantly different than after oral feeding. When examining the metabolite functional enrichment between time points, several differential metabolic pathways were identified. For example, in the saliva samples, vitamin B5 CoA biosynthesis and glutathione metabolism changed significantly after oral feeding. Vitamin B5 is a water-soluble vitamin and a precursor in the synthesis of CoA, which is essential to many biochemical reactions to sustain life including fatty acid and bile acid synthesis. Glutathione has important roles in nutrient metabolism, antioxidant defense, and regulation of cellular events such as immune response, cell proliferation, and apoptosis. Deficiency of glutathione contributes to oxidative stress which is linked to the pathogenesis of several diseases such as liver disease, cancer, and diabetes.^49^

In the milk samples, several differential metabolic pathways were identified as having changed when comparing the samples before and after oral feeds. These included Vitamin E metabolism, Vitamin B12 metabolism, and Tryptophan metabolism. Vitamin E is an important antioxidant and an essential vitamin in infants; its deficiency has been linked to the development of intraventricular hemorrhage, bronchopulmonary dysplasia, edema, hemolytic anemia, central nervous system development delay, and cardiomyopathy.^50^ Vitamin B12 is a regulator of fetal growth and plays an important role in cellular metabolism. Its deficiency can cause significant issues in infants including failure to thrive, neurological and cognitive dysfunction, and hematological problems.^50^ Lastly, Tryptophan is an essential amino acid, and in breastfed infants, is the only amino acid source available in HM. According to the literature, the implications of Tryptophan deficiency in infants is unclear but is thought to have potential neurological implications in infants.^51^

Previous studies have demonstrated that metabolite concentrations, mainly carbohydrates and amino acids, change with the duration of lactation.^52,53^ Higher levels of amino acids are found in colostrum, whereas higher levels of saturated and unsaturated acids are found in more mature milk.^54^ Differences in metabolite concentrations have also been noted when comparing the milk of preterm to term infants,^52^ across specific geographical locations,^55^ and in varying pathophysiologic diseases of mothers (such as gestational diabetes).^54^ Further investigation is warranted as it may provide insight into the preterm infant’s overall health and risk for adverse outcomes. Furthermore, it would be of interest to further examine the relationship between the microbiome-metabolome which could also be used as a biomarker of disease risk in preterm infants.

There were several strengths to our study design. This appears to be the first study examining the impact of the transition from NG to oral feedings on the preterm infant’s microbiome and metabolome. The study collected extensive maternal and infant clinical information that related to the analysis of the samples (Supplementary Table 1). Clinical factors such as age at time of sample, sex, twin status, birth weight, antibiotic use, maternal chorioamnionitis, and number of days intubated were examined, however, the low sample size and unique clinical history of each participant did not result in any statistical significance of these factors.

We also had several limitations to our study. The sample size of our cohort was small with only 11 maternal-infant dyads. We were further restricted due to the preterm infant’s intestinal microbiota which is known to have a delayed colonization of microbes and overall low biodiversity.^56,57^ This likely hindered our ability to identify any changes in the stool microbiome after initiating oral feeds. Furthermore, HM itself has a very low biomass at baseline,^58–60^ and insufficient DNA yield and read depth from some samples further restricted our resulting sample size. While we were able to detect DNA within the stool, saliva and milk samples, it does not indicate the presence of viable or even intact bacteria.^61^

Additionally, feeding of preterm infants is very individualized and the composition of what they receive enterally changes often and is based on HM availability, GA, fortification needs, and stability of the patient. The intent of the MAP study was to collect milk samples of what the infant was feeding at that moment in time (whether it included donor milk, fortifier, or exclusively formula, etc.). As such, the HM samples available were variable (Supplementary Table 2). Previous studies have shown that fortifiers have a significant effect on the fecal microbiota composition in preterm infants.^5,14,62^ Another study found that the source of HM (mother versus donor) had a more significant effect on the preterm infants microbiome than the fortifier type.^63^ Additionally, a study by Moossavi et al. found that providing pumped HM was consistently associated with the enrichment of potential pathogenic bacteria and reduction in *Bifidobacteria* in HM, compared to when directly breastfeeding.^41^ Once oral feeds were started in our cohort, there was variability in whether and how often infants were bottle feeding versus direct breastfeeding. These factors made it difficult to stratify the samples into groupings to statistically compare, both within each dyad and across the cohort. Also, there were several confounding factors that have not been accounted for including non-nutritive breastfeeding, current maternal diet/medications, etc. due to lack of clinical data available.

## Conclusion

In conclusion, our study demonstrated potentially beneficial changes in the HM and infant saliva microbiome and metabolome over the first 4–5 months of life for preterm infants, particularly after oral/breastfeeding initiation. Larger cohorts are needed to determine whether these changes can be used as biomarkers to determine disease risk.

## Supplementary Information

Supplementary Figures

## Data Availability

Metagenomic raw sequence files and metadata tables are publicly available in the NCBI Sequence Read Archive (SRA; BioProject ID PRJNA1086674). Metabolomic raw data, mzXML files spectral files (.mgf), and feature quantification tables can be found on the MassIVE (https://massive.ucsd.edu/) database, under the accession numbers MSV000090521 (Stool samples), MSV000090522 (Saliva samples), and MSV000090523 (Milk samples).
