# Supplementary material for: The impact of breastfeeding on the preterm infant’s microbiome and metabolome: a pilot study
Source: Pediatr Res. 2024 Aug 13;97(3):1227–36. doi: 10.1038/s41390-024-03440-9 (PMC12055563; doi:10.1038/s41390-024-03440-9)
Supplement: Supplementary file 1 — Supplementary Figures [file 41390_2024_3440_MOESM1_ESM.pdf]

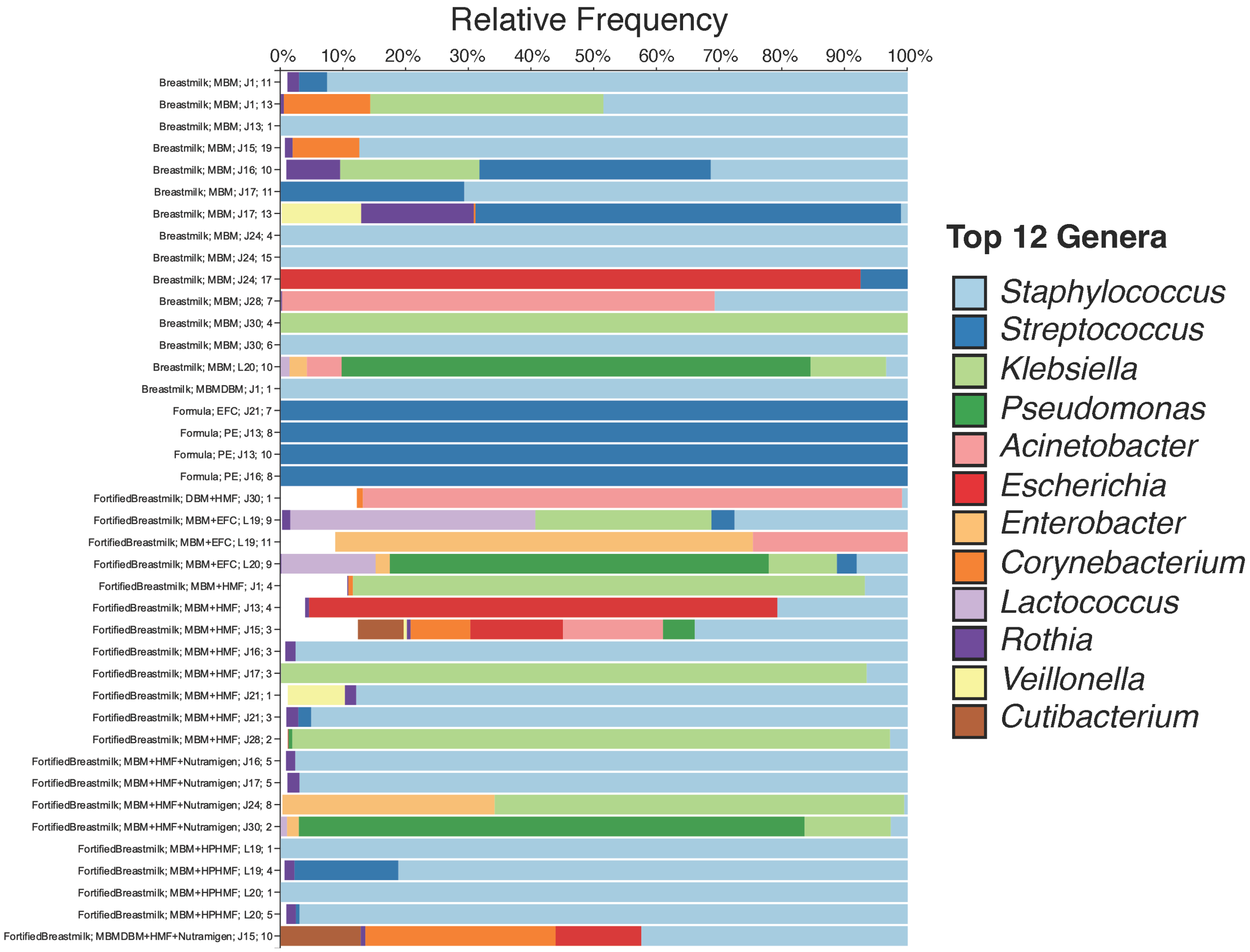

Supplementary Figure 1: Genera in milk samples, organized by milk type, participant, and time point. Top 12 most relatively abundant bacterial genera identified are shown, with the milk dataset rarefied to 1,770 sequences per sample. The remaining other taxa are grouped in white.

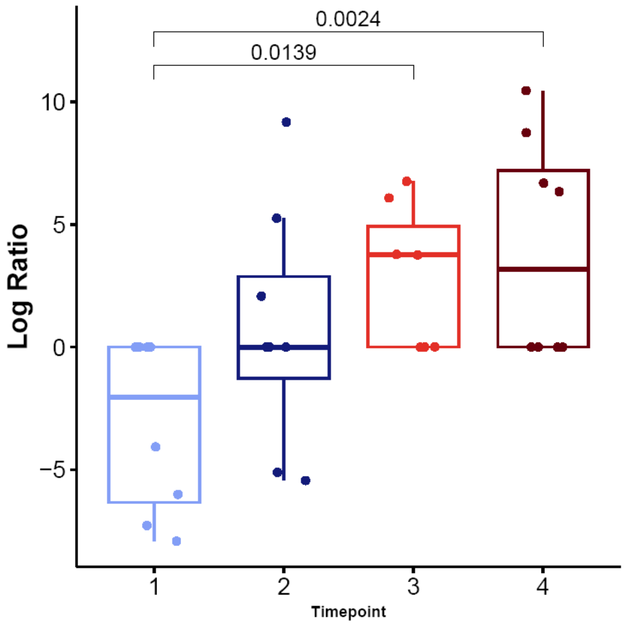

Supplementary Figure 2: Milk samples. Box plots of log ratio of top 20 bacterial operational genomic units (OGUs) to bottom 20 OGUs associated with time points 1 and 4, plotted across all time points. Log ratios were calculated and processed using Songbird multinomial regression and visualized in Qurro. Statistical significance was determined by linear mixed effect models, LME, $p<0.05$.

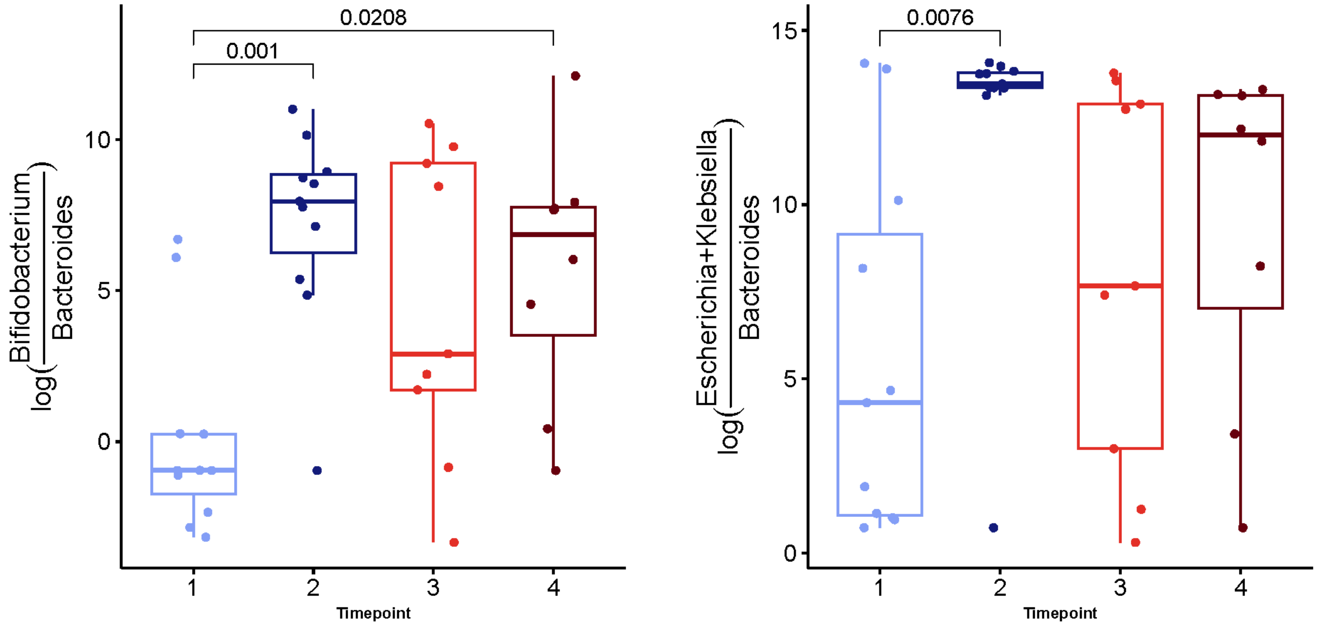

Supplementary Figure 3: Stool samples. Box plots of log ratios of all of the OGUs identified within the genera *Bifidobacterium/Bacteroides* and *Escherichia* and *Klebsiella/Bacteroides*. Log ratios were calculated and processed using Songbird multinomial regression and visualized in Qurro. Statistical significance was determined by linear mixed effect models, LME, $p<0.05$.

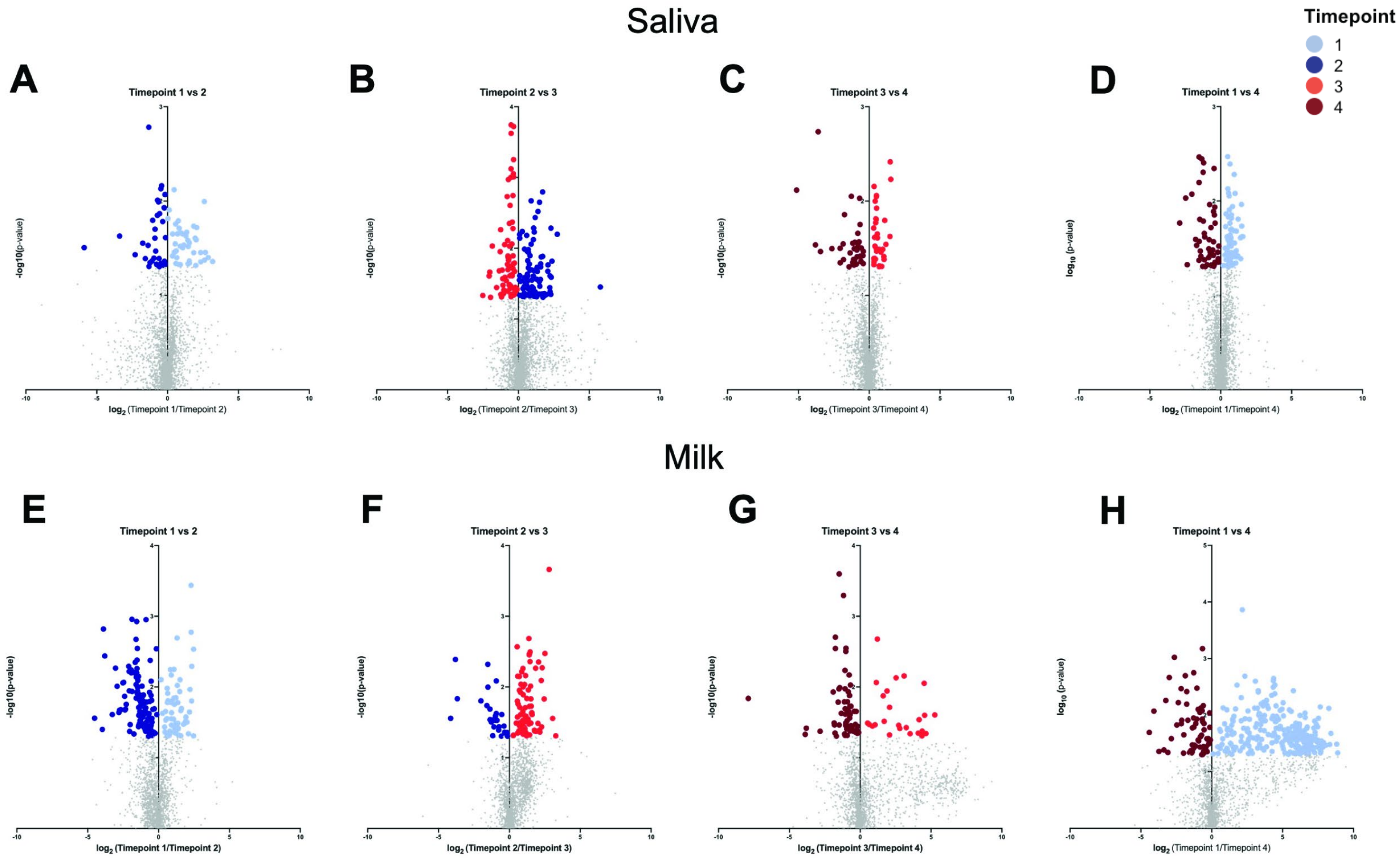

Supplementary Figure 4: GNPS annotated features were significantly altered by time point. (A-H) Volcano Plots demonstrating binary comparisons of saliva and milk samples by time points. Grey points represent metabolite features with no significant difference ($p>0.05$ Student's t test with Benjamini-Hochberg adjusted values). Significantly altered metabolites are colored by time point in which they are found to be higher in each respective binary comparison.

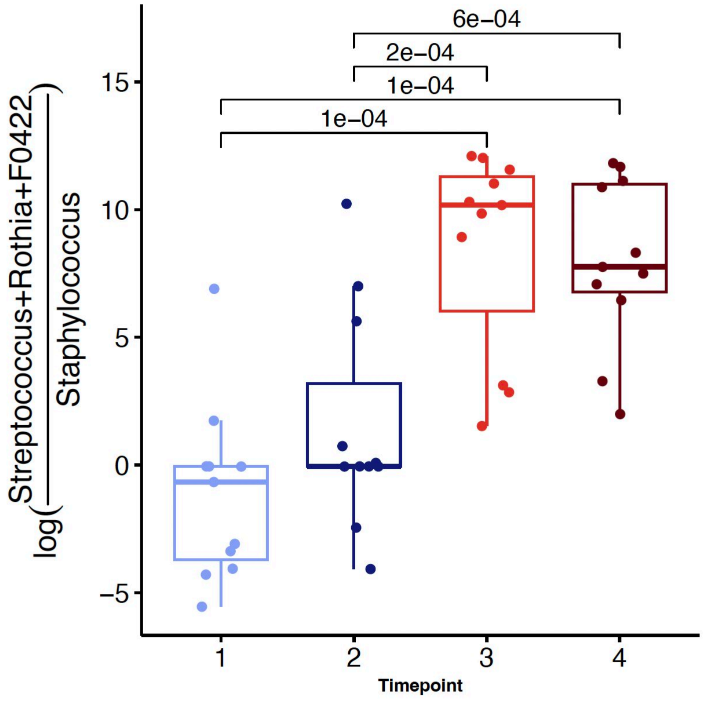

Supplementary Figure 5: Saliva samples. Box plots of log ratio of all of the OGUs identified within the genera *Streptococcus*, *Klebsiella* and *Veillonella F0422* to those within *Staphylococcus*. Log ratios were calculated and processed using Songbird multinomial regression and visualized in Qurro. Statistical significance was determined by linear mixed effect models, LME, $p<0.05$.

Supplementary Table 1: Infant demographics, birth history, and NICU Course.

Characteristics	Maternal-Infant dyads n=11
Gestational Age, average weeks (range)	27.9 (23.4 – 32.2)
Male infants, n (%)	4 (36%)
Number of infants who are a twin, n	5
Pair of twins, n	2
Average weight at birth, g (range)	1113 (575 – 1855)
Average birth length, cm (range)	36 (30.5 – 44)
Average head circumference, cm (range)	26 (21 – 31)
Median APGAR at 1 min, (range)	4 (1 – 8)
Median APGAR at 5 min, (range)	8 (3 – 9)
Median APGAR at 10 min, (range), n = 4	6.5 (6 – 7)
Number of infants who were intubated, n (%)	7 (64%)
Average intubation duration, days (range), n = 7	8 (1 – 23)
Surfactant administration, n (%)	7 (64%)
Number of infants who received a systemic steroid, n (%)	2 (18%)
# of infants exposed to postnatal antibiotics > 2 days, n (%)	8 (73%)
# of infants exposed to postnatal antifungal, n (%)	2 (18%)
Number of infants required antacid, n (%)	1 (9%)
Infants' day of life when enteral feeds started, # (range)	1 (0 – 3)
Infants' day of life when oral feeds started (breast or bottle, whichev	42 (19 – 75)
Infants' day of life when breast feeds started, # (range)	47 (24 – 85)
Infants' day of life when bottle feeds started, # (range)	48 (19 – 98)
Duration of NICU hospitalization, # days (range)	86 (49 – 146)
Diet at discharge	
Anti-reflux formula, n (%)	2 (18%)
Preterm formula, n (%)	1 (9%)
Preterm formula and HM, n (%)	6 (55%)
Maternal HM (supplemented with formula for increased calories)	2 (18%)
Complications	
PDA requires treatment, n	2 (18%)
ROP, n	2 (18%)
IVH:	
Grade 1 and Grade 2, n	6 (55%)
Grade 3 and Grade 4, n	0 (0%)
Necrotizing enterocolitis, n	0 (0%)
SIP, n	0 (0%)
Home oxygen, n	2 (18%)
G-tube, n	1 (9%)

Abbreviations: ROP: retinopathy of prematurity, PDA: patent ductus arteriosus, G-tube: gastrostomy tube.

Supplementary Table 2: Composition of HM samples collected.

**Type of milk samples**	**# of samples, n=43**
Maternal HM	15
Maternal HM + Fortifier*	21
Maternal HM / Donor HM	1
Donor HM + Fortifier*	1
Maternal HM / Donor HM + Fortifier*	1
Formula	4

*Fortifiers used includes: Enfacare, HM Fortifier (HMF), HMF-HP (Hydrolyzed Protein), Nutramigen

Supplementary Table 3: Average weight gain during NICU admission.

	**Average daily Wt gain (g) prior to PO feeds, n=11**	**Average daily Wt gain (g) from initiation of PO to full PO feeds, n=9***	**Average daily Wt (g) change from full PO feeds to discharge, n=9***	**Average daily Wt (g) change during admission, n=11**
Average	18.7	35.3	26.8	26.6

*One infant was transferred to another facility prior to reaching full oral feeds, another infant never achieved full oral feeds and received a gastric tube prior to discharge.
